# Supplementary material for: Decoding ultrasensitive self-assembly of the calcium-regulated Tetrahymena cytoskeletal protein Tcb2 using optical actuation
Source: J Biol Chem. 2025 Oct 16;301(12):110824. doi: 10.1016/j.jbc.2025.110824 (PMC12661445; doi:10.1016/j.jbc.2025.110824)
Supplement: Supplemental Figures [file mmc5.pdf]

Supporting information for:

Decoding ultrasensitive self-assembly of the calcium-regulated *Tetrahymena* cytoskeletal protein Tcb2 using optical actuation.

Nithesh P. Chandrasekharan[1,2], Xiangting Lei[3], Jerry Honts[4], Saad Bhamla[3], Scott M. Coyle[1,*]

Affiliations:
[1]Department of Biochemistry, University of Wisconsin-Madison
[2]Integrated Program in Biochemistry Graduate Program
[3]School of Chemical and Biomolecular Engineering, Georgia Institute of Technology
[4]Department of Biology, Drake University

University of Wisconsin-Madison, Madison, Wisconsin 53706, USA.
*Correspondence to: smcoyle@wisc.edu

Contains:

Supplemental figures 1-4.

Supplemental movie legends 1-4.

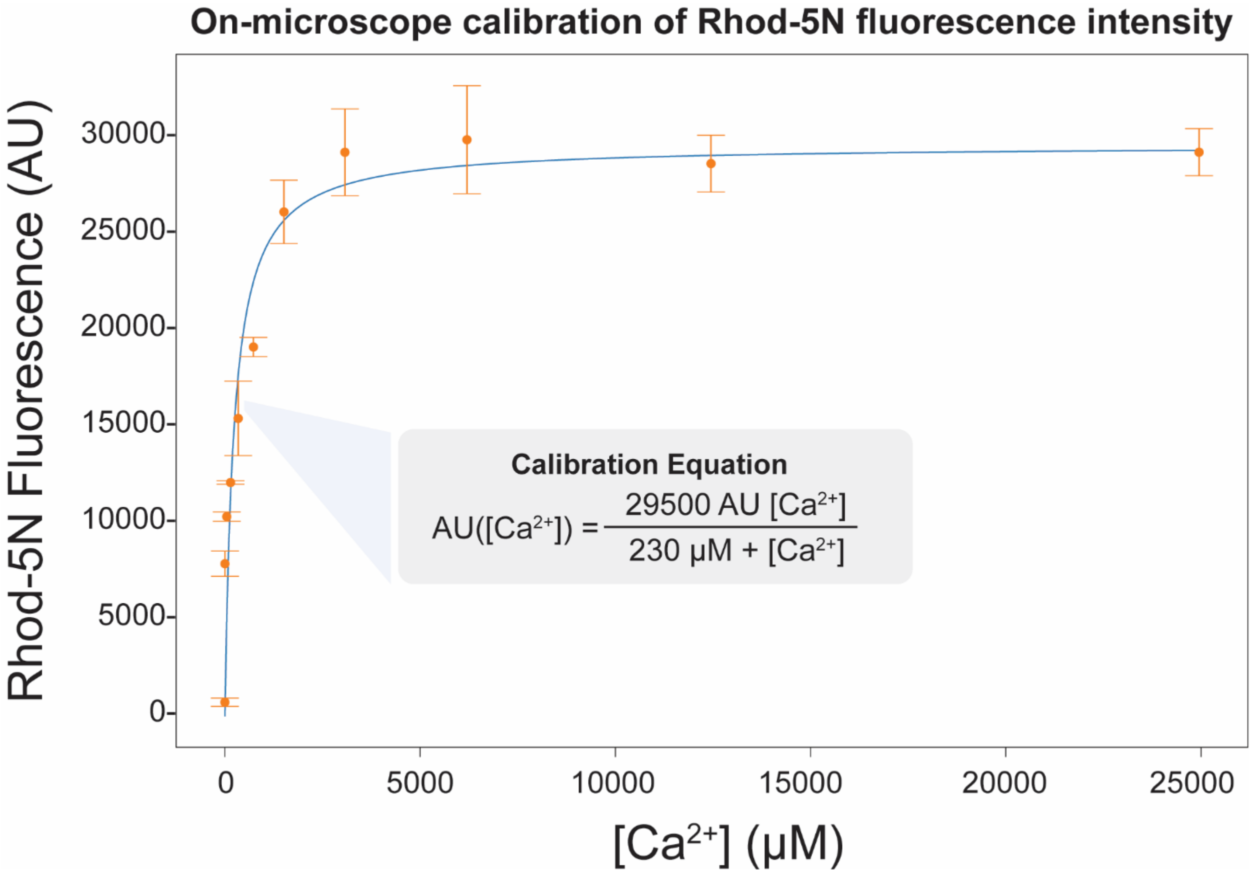

Supplemental Figure 1. Sample calibration curve equation determine fitting standards of 2μM to 25mM [Ca^{2+}] in the presence of 25uM Rhod-5N imaged with our optical set up. Standards are fitted to a binding curve ($y=A*x/K_D + x$) and rearranged to make a calibration curve that converts Rhod-5N intensities into calcium concentrations. All points represent mean values derived from ≥ 3 independent biological replicate preparations of the indicated calcium concentration, with N ≥ 5 independent technical replicate fluorescence measurements per replicate sample. Error bars denote standard deviation across the biological replicates.

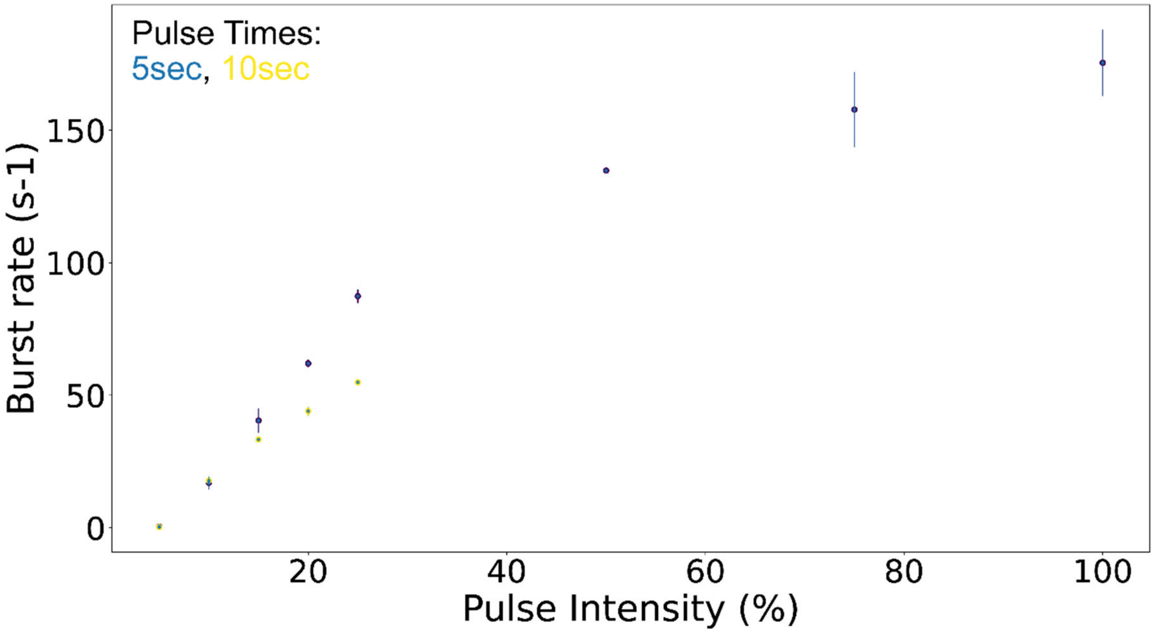

Supplemental Figure 2: Rate of burst phases scales with intensity

All data points represent mean burst rate values derived from ≥ 3 independent biological replicate samples, with N ≥ 5 independent technical replicate pulses per sample. Error bars denote standard deviation across the biological replicates.

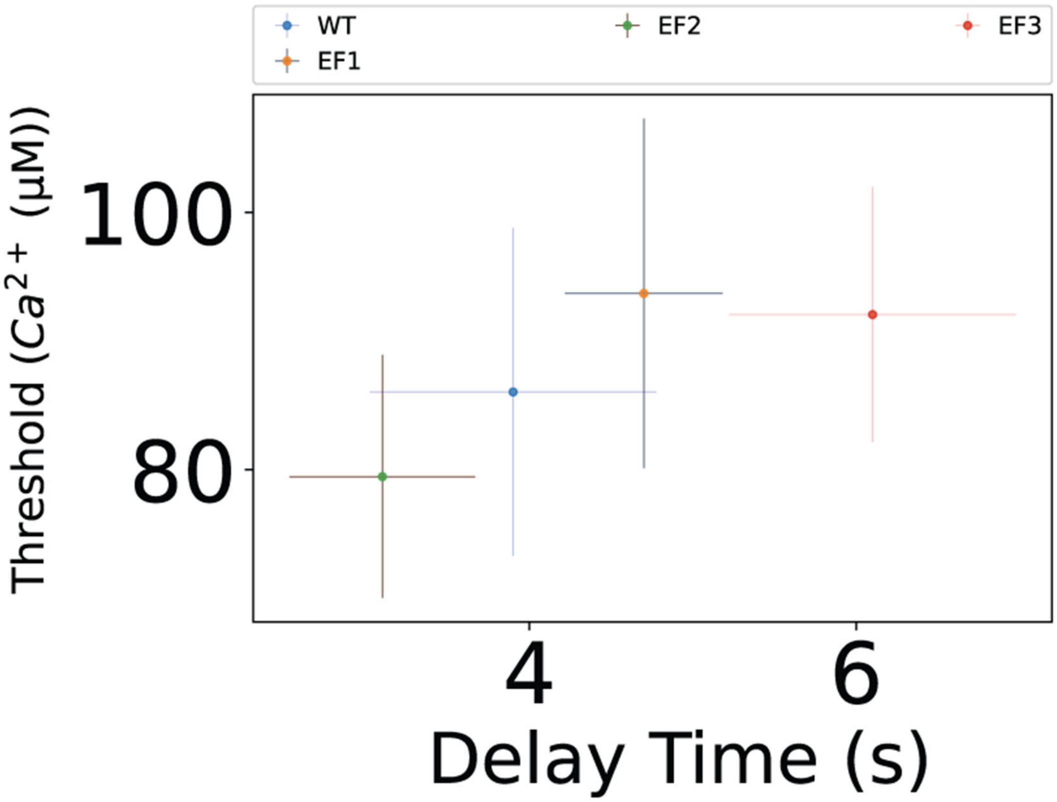

Supplemental Figure 3. Correlation between [Ca^{2+}·Tcb2] threshold and time delay in triggering Tcb2 self -assembly among the EF-hand knockouts. Lowered threshold weakly correlates with a faster time delay while a higher threshold indicates a longer time delay. Points denote the mean delay time and threshold [Ca^{2+}] values derived from ≥ 3 independent biological replicate samples of the indicated protein, with N ≥ 5 technical replicate pulses per sample. Error bars denote standard deviation for the delay time (x-axis) and threshold [Ca^{2+}] concentration (y-axis) across the biological replicates.

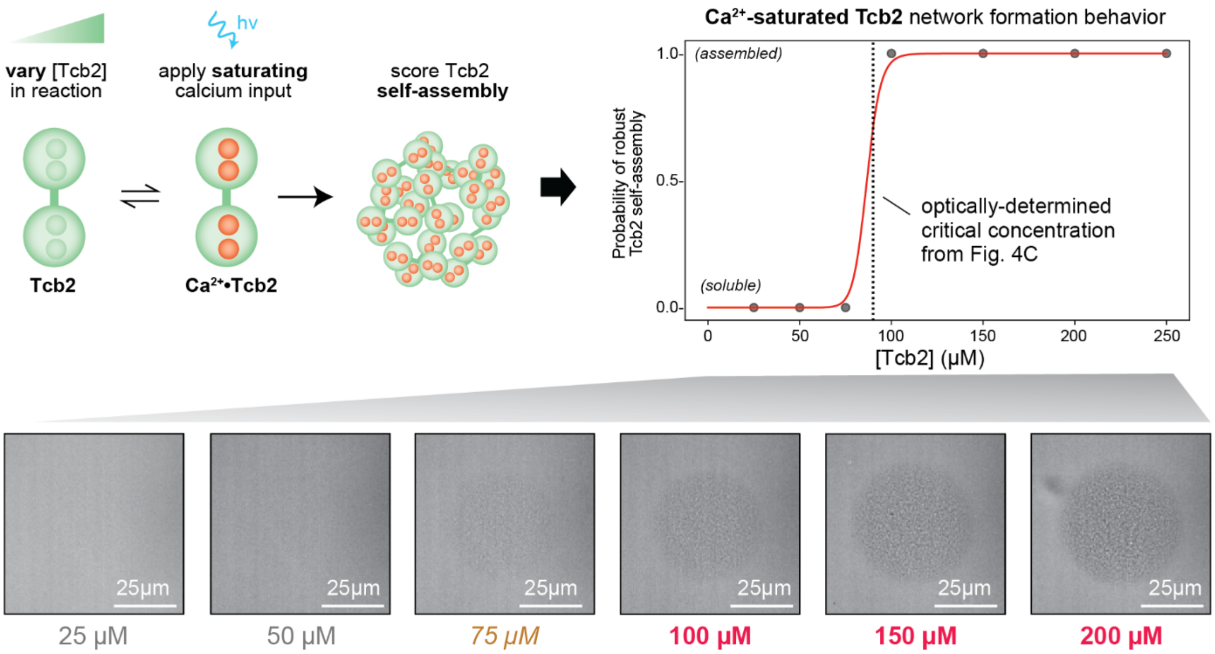

Supplemental Figure 4. Tcb2 self-assembly behavior at different protein concentrations under saturating calcium loading conditions. Tcb2 solutions were prepared over a range of different concentrations, and a saturating calcium input was applied to the sample (100%, 1s intensity light pulse). Self-assembly was scored as in Fig. 4C (see Methods) for N=5 biological replicates with 1 technical replicate pulse each and plotted as a function of [Tcb2]. Representative images post-stimulation for concentrations < 250 μM are shown.

Supplemental movie legends.

Movie S1.
Example demonstration of optically controlled triggering of Tcb2 self-assembly. A digital mirror device (DMD) attached to a microscope is used to project a controlled, micron scale pulse of UV light pattern onto a concentrated Tcb2 solution. Upon light stimulation, Tcb2 networks immediately become visible by brightfield microscopy in and around the area of stimulation and cease to expand radially when the light is turned off. See also main text Fig. 1.

Movie S2.
Examples videos showing identical Tcb2 solutions optically stimulated with different pulse parameters producing different responses: a small 25 um ROIs with long 10s pulse; and a large 200 um ROI with a short 1s pulse. See also main Fig. 2.

Movie S3.
Example video showing Rhod-5N fluorescence during optical actuation in the presence or absence of Tcb2. The spatiotemporal Rhod-5N intensity profile decreases everywhere in the field of view in the presence of Tcb2 via competition for released calcium. A brightfield image of Tcb2 network formation occurring in parallel during the pulse is also shown. See also main text Fig. 4.

Movie S4.
Comparison of network formation for wildtype Tcb2 or EF-hand point mutants in response to an identical high-intensity optical stimulation protocol. While WT, EF1mut, EF2mut, EF3mut all formed networks, no network formation was observed for the EF4mut. See also main text Fig. 5.
